# Compassion Focused Therapy to Address Shame and Guilt: A Case Study of a Client With Complex PTSD

**DOI:** 10.1002/jclp.70145

**Published:** 2026-04-28

**Authors:** Deborah Lee, Dorothy King, James N. Kirby

**Affiliations:** ^1^ Berkshire Traumatic Stress Service, Berkshire Healthcare NHS Foundation Trust, Reading Berkshire UK; ^2^ The Compassionate Mind Research Group, The School of Psychology The University of Queensland Brisbane Australia

**Keywords:** compassion focused therapy, CPTSD, guilt, shame, trauma

## Abstract

This paper presents the case of “Ava” a woman in her late 40s diagnosed with ICD‐11 Complex Posttraumatic Stress Disorder (CPTSD), whose life was shaped by chronic childhood abuse, pervasive shame, and intense self‐criticism. Ava struggled with intrusive trauma memories, relational hypervigilance, fears of compassion, and enduring guilt regarding the impact of past substance misuse on her children. A phased Compassion Focused Therapy (CFT) approach was implemented to address her chronic threat state (persistent hypervigilance and relational alarm) and facilitate a shift from shame‐based self‐attack toward compassionate self‐relating. Phase 1 involved 12 group sessions focused on developing compassionate resilience and social safeness. Phase 2 consisted of 28 individual sessions integrating trauma memory processing with compassion focused interventions. Phase 3 included an optional, pilot 8‐session group, consolidating compassionate identity and values‐based living. The therapeutic journey involved relational challenges, including fears of judgment, ambivalence about relinquishing self‐criticism and moments of alliance strain. Over the course of treatment, Ava demonstrated clinically significant reductions in CPTSD symptoms and trauma related shame, alongside increased self‐reassurance, emotional regulation, and consolidation of a compassionate identity. Clinical implications of trauma focused CFT for those with complex PTSD are discussed.

## 1 Introduction

Compassion Focused Therapy (CFT) is an evolutionary informed, biopsychosocial and contextual model of therapy developed by Gilbert (Gilbert and Simos 2022). It aims to directly target the experiences of shame and self‐criticism, both of which significantly impact mental health (Löw et al. 2020). A growing number of meta‐analyses indicate CFT improves mental health in both clinical and non‐clinical populations (e.g., Petrocchi et al. 2024). CFT is informed by social mentality theory, which proposes that evolved motivational systems influence patterns of cognition, affect, physiology, and behavior, particularly within relational contexts (Gilbert and Simos 2022).

Shame is a multifaceted experience involving perceptions of social devaluation or inferiority. It may arise externally (beliefs that others view the self negatively) or internally (harsh self‐evaluation; Gilbert and Andrews 1998). Within CFT, shame is understood as linked to competitive and rank‐based motivational systems. When individuals perceive themselves as inferior or socially threatened, this may trigger submissiveness, avoidance, or internal self‐criticism as an attempt to manage perceived threat (Gilbert 2014). However, persistent self‐criticism is associated with poorer mental health outcomes and reduced responsiveness to treatment (Löw et al. 2020). In contrast, guilt is linked to compassionate motivation. Rather than reflecting global self‐condemnation (“I am bad”), guilt involves specific appraisal of behavior (“I did something bad”) and can motivate repair and reconnection (Lee et al. 2001). However, in trauma‐related contexts, guilt may become chronic and fused with shame, contributing to rumination and psychopathology, such as low mood and PTSD (Lawrence and Lee 2014).

Within a CFT framework, shame is considered a primary response to social threat, and self‐criticism as a secondary safety strategy (Gilbert and Andrews 1998), and both emerge in response to chronic relational threat and attachment alarm. Individuals with complex trauma histories often live in persistent states of interpersonal hypervigilance, anticipating rejection, criticism or abandonment. Accordingly, CFT seeks to reduce chronic threat activation and cultivate compassionate motivational systems capable of regulating relational fear. However, the process of motivational shifting can be difficult, particularly as individuals high in shame can experience fears, blocks and resistances to compassion (Kirby et al. 2019). Indeed, a mixed‐methods study with those with PTSD found that many believed they did not deserve compassion because their “illness” was their own fault (Lawrence and Lee 2014). Fears of compassion are particularly prevalent in individuals with histories of childhood trauma (Matos et al. 2023). CFT uses practices such as grounding, soothing rhythm breathing, compassionate imagery and compassionate self‐development (Petrocchi et al. 2024). Introducing compassion practices without sufficient validation and de‐shaming may activate threat responses and increase disengagement (Matos et al. 2023). Therefore, addressing fears of compassion is a central therapeutic task in CFT.

CFT has been applied across several trauma‐exposed populations, including survivors of sexual abuse (McLean et al. 2022) and veterans with PTSD (Romaniuk et al. 2023). Emerging qualitative and single‐case experimental research on CFT‐informed group interventions for complex trauma suggests improvements in shame, social safeness, and emotion regulation. This paper extends this work by presenting a detailed case illustration of a woman with ICD‐11 CPTSD, chronic shame and guilt, and fears of compassion, treated using a phased CFT model delivered across group and individual formats.

### 1.1 A Compassion Focused Perspective on Complex PTSD

ICD‐11 (World Health Organization 2022) describes Complex PTSD (CPTSD) as arising following exposure to prolonged or repeated traumatic events, often extremely threatening, horrific and interpersonal in nature. In addition to core PTSD symptoms (re‐experiencing, avoidance, hyperarousal), CPTSD includes disturbances in self‐organisation (DSO): affect dysregulation, persistent negative self‐concept, pervasive shame or guilt, and interpersonal difficulties. These difficulties commonly arise in the context of chronic relational trauma, particularly where caregivers are sources of harm (Farina et al. 2025).

Within a CFT framework, CPTSD can be understood as involving persistent activation of relational threat systems (Cloitre 2019) often arising from chronic interpersonal trauma and attachment disruption (Karatzias et al. 2017). Such developmental environments prime individuals toward chronic activation of threat and rank‐based motivational systems. Individuals may live in states of heightened vigilance to social danger, anticipating rejection, criticism, or abandonment. Shame, guilt, and harsh self‐criticism frequently emerge within this context as threat‐management strategies aimed at minimizing perceived interpersonal risk. For instance, when attachment figures are frightening, neglectful, or inconsistent, children may adopt submissive, appeasing, or self‐blaming strategies to minimize danger. This can include taking responsibility for perceived “wrongdoing” when subjected to abuse from caregivers. Over time, these adaptations may consolidate into enduring self‐critical relational styles and heightened sensitivity to social threat. Underdevelopment of compassionate motivational systems may leave individuals with limited capacity for self‐soothing, increasing reliance on avoidance strategies such as substance use (Popolo et al. 2024).

CFT psychoeducation frames self‐criticism as an evolved threat‐protection response rather than a personal flaw. Explaining that CPTSD symptoms reflect adaptations to chronic attachment threat can help de‐shame clients’ experiences and foster compassion toward the self (Steindl et al. 2023). CFT aims to help individuals recognize these patterns as understandable adaptations to relational threat and gradually cultivate compassionate motivational systems capable of regulating fear, shame and interpersonal alarm. CFT is theoretically well aligned with ICD‐11 CPTSD, particularly the disturbances in self‐organisation. Treatment therefore aims to facilitate motivational shifting from threat‐based self‐relating toward compassionate engagement with distress. It aims to improve self‐to‐self relating, interpersonal functioning and emotion regulation.

### 1.2 A Phased Approach to Treating CPTSD With CFT

**Figure 1:**
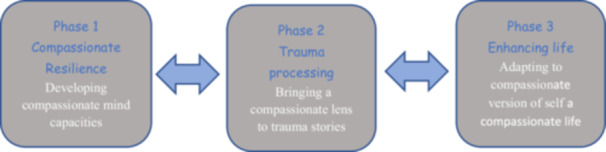
The phased treatment approach CFT for CPTSD.

CFT for CPTSD can be conceptualized as a phased approach to treatment, consistent with ISTSS best‐practice recommendations for complex trauma: stabilization‐trauma processing‐ reclaiming life, (Cloitre 2019). In line with this, the intervention first emphasizes CFT *de‐shaming* psychoeducation, the cultivation of compassionate motivation, stabilization and safeness through emotion regulation capacities and self‐compassion before engaging in trauma memory processing, followed by consolidation of compassionate identity and adaptation to present life circumstances. Although described sequentially, movement between phases is formulation‐driven rather than strictly linear. Therapists may return to earlier processes in response to shifts in presentation, emotional tolerance, or relational dynamics. Figure 1 summarizes the three phases of treatment.

#### 1.2.1 Phase 1: Developing Compassionate Resilience

This phase focuses on CFT *de‐shaming* psychoeducation, cultivating compassionate motivation and strengthening the client's capacity for *internal safeness*. This refers to developing a felt sense of warmth, care and affiliative regulation (Gilbert and Simos 2022), rather than relying primarily on threat‐based coping strategies associated with *safety* (the removal of threat). This phase draws particularly on processes of awareness, differentiation, tolerance, and integration.

**Figure 2:**
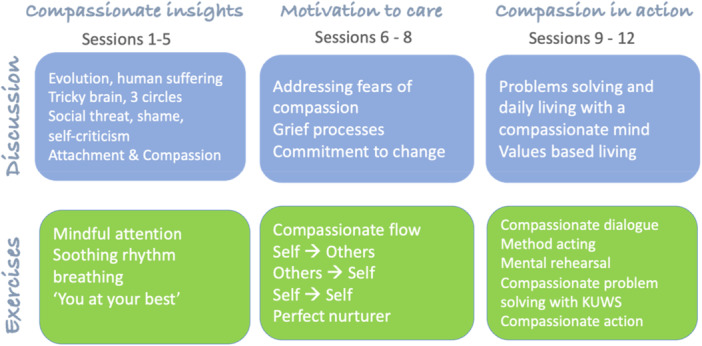
Compassionate resilience 12‐session group.

CFT psychoeducation is presented didactically (via workbook and presentation), then explored through Socratic questioning and guided discovery. Clients are supported to understand the evolutionary origins of threat responses and self‐criticism, to help lessen the intensity of shame and self‐blame. In this case, phase 1 was delivered as a 12‐session closed online group. Each participant is given a workbook to accompany the group with key psychoeducation, exercises and practice sheets. Group delivery can enhance social safeness, common humanity and affiliative motivation, while also providing opportunities to work directly with social rank sensitivities as they emerge interpersonally (Matos et al. 2017). However, group contexts may also activate fears of comparison or judgment, requiring careful facilitation. In some cases, and therapy settings where a group is not feasible, the group content can be adapted for individual therapy. The group introduced a structured compassionate framework incorporating compassionate knowledge (de‐shaming psychoeducation), understanding (empathy and insight into developmental origins), wisdom (balanced and caring perspectives), and strength (courage to alleviate suffering). Figure 2 outlines the group structure and core exercises.

#### 1.2.2 Phase 2: Trauma Processing

Phase 2 integrates trauma memory processing within a compassionate framework. Drawing on processes of integration and new contextual learning, clients engage in narrative reconstruction, imagery rescripting, chair work, and reliving techniques. The aim is not only symptom reduction, but transformation of shame‐based meanings associated with traumatic events. Transition to this phase was guided by improvements in Ava's emotional regulation, access to compassionate states, reduced fusion with self‐criticism and increased tolerance of relational threat. The number of sessions varies according to formulation and trauma complexity. Typically, this phase requires 16–24 individual sessions delivered either face to face or online (dependant on client preference and risk). In this case, phase 2 included 28 individual sessions due to the intensity of self‐blame and fears of compassion.

#### 1.2.3 Phase 3: Enhancing Life and Future Orientation

The final phase consolidates compassionate identity and supports adaptation to present and future life circumstances. Drawing on processes of cultivation, transformation and embodiment, clients are encouraged to align behavior with values and compassionate intention. This may involve behavioral experiments, values‐based planning and method acting techniques to strengthen compassionate self‐identity. In this case, phase 3 was delivered as an 8‐session pilot group focused on relapse prevention and sustaining compassionate self‐relating. Progression to this phase was informed by reductions in re‐experiencing and avoidance, improved affect regulation, and greater stability in self‐to‐self relating. Across all phases, the model remains recursive. Therapists may return to earlier processes when fears of compassion re‐emerge or when trauma processing activates threat responses. The phased structure therefore serves as a scaffold within a flexible, formulation‐driven approach. We now present a case illustration of Ava, using this phased CFT approach.

## 2 Case Illustration

### 2.1 Presenting Problem and Client Description

Ava, a white woman in her late forties, was referred to the Traumatic Stress Service for Compassion Focused Trauma Therapy (CFT‐Tr) to treat her CPTSD, anxiety, and depression. She had a history of self‐harm, alcohol/substance abuse and was previously involved with various mental health services, including a 2‐year period at a therapeutic day unit. She had not used alcohol or self‐harmed for 18 months prior to current treatment.

Ava grew up in a stressful, unstable, and fearful home environment, marked by persistent tension. Her childhood was defined by multiple forms of abuse (physical, emotional, sexual, neglect) within the family, as well as chronic bullying at school. Medical professionals noted her “nervous disposition” and “failure to thrive” early on, which impacted her self‐perception. She felt constantly compared unfavorably, to her four siblings and cousins, which made her feel inadequate. She described her mother's punishments as “disproportionate to the act” and her father as, unpredictable and volatile. This led her to develop coping mechanisms such as withdrawal and people‐pleasing to avoid conflict.

Her parents separated when she was 3‐ to 4‐years old, and when she was about 5‐years old, Social Services became involved with the family, because of her father's alcohol use. Thereafter, he had restricted access to his children. The family experienced financial hardship but managed to have some positive experiences, due to financial support from Ava's grandmother and an aunt, for holidays and Christmas. She remembered that 1 year, a charity donated Christmas presents.

During later childhood, she experienced sexual abuse from a neighbor and a family friend. Ava experienced her mother's “protective” response to the abuse perpetrated by the family friend (i.e., threatening the perpetrator) frightening rather than supportive. Her mother never showed affection, but would often react, and overreact, aggressively. Ava did not feel able to disclose the further abuse by the neighbor, for fear of her mother's response.

School was challenging due to bullying and feeling like an outsider. As a teenager, she sought validation and praise by constantly volunteering to do things around the home so that her mum would think she was helpful. In her late teens, she began using drugs and alcohol to manage emotional distress and intrusive memories/flashbacks to the abuse. She continued to misuse substances throughout her early 20s, briefly interrupted by military service and her first pregnancy. However, her use escalated after a combination of life events: the birth of her third child, the death of her mother, a house move, and the increasing realization of her difficult childhood. She also began cutting at this time.

The breakdown of her marriage led to continued heavy substance use and involvement with Social Services. Her children temporarily moved in with their father as she could not manage. Drug dealers frequented the house. She later realized that these acquaintances had provided her with a sense of validation as they made her feel like she was a “someone.” Throughout these times she kept her job at an electronics factory and was highly regarded. Ava ultimately stopped her substance misuse but this triggered a flooding of trauma memories and flashbacks, which led her to seek help. She now has good, supportive relationships with her four children, but she struggles with strong feelings of guilt about the impact of her past substance use on her family.

### 2.2 The Therapists

Dr X is a Consultant Clinical Psychologist and has been qualified for 35 years. She has specialized in the treatment of PTSD and CPTSD throughout her career, and in adapting the Compassion Focused Therapy for CPTSD. Dr X was the primary group facilitator for the compassionate resilience phase of Ava's treatment. Dr Y is a Principal Clinical Psychologist who has been qualified for 7 years. She specializes in treating CPTSD with CFT. Dr Y has specialized in CFT for 6 years and has recently been a trial therapist in a national RCT investigating phased treatments for CPTSD. Dr X supervised this work.

### 2.3 Case Formulation

**Figure 3:**
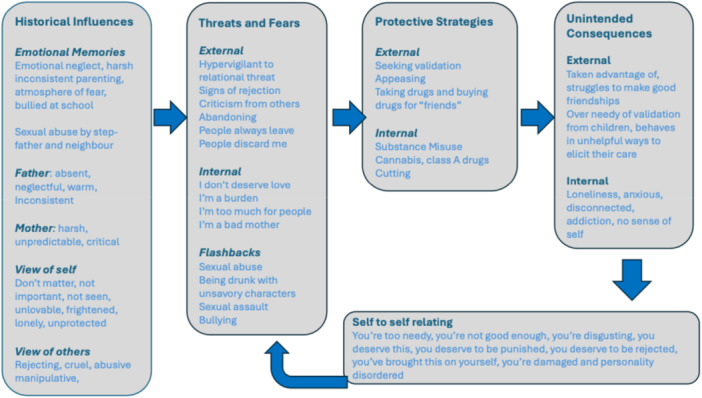
Ava's CFT case formulation.

CFT formulates cases from an evolutionary, biopsychosocial model (Gilbert and Simos 2022). Figure 3 summarizes the key aspects of Ava's formulation, linking internal and external threats to social relating which is explored more fully below.

The formulation is relational and motivationally informed, focusing on how evolved threat, drive, and affiliative systems interact within the client's developmental history. Attention is given to key emotional threats (e.g., rejection, abandonment, humiliation), the protective strategies that emerge in response to these threats, and the unintended consequences that maintain distress. Formulation maps how self‐to‐self and self‐to‐other relating become organized around chronic relational alarm and rank‐based concerns, and it guides the pacing and sequencing of therapeutic intervention within the phased model.

#### 2.3.1 Historical Influences

Ava's development was profoundly shaped by a fragmented and unsafe family environment following her parents’ early separation and her father's limited presence. This contributed to a lack of secure attachment and a deep sense of vulnerability, evidenced by an early diagnosis of a “nervous disposition” and “failure to thrive” requiring multiple hospitalizations. She was bullied at school, her childhood was marked by severe emotional neglect, harsh and inconsistent discipline from her mother, and exposure to various forms of abuse (emotional, physical, and sexual) from a neighbor and from a family friend. Although Social Services were involved, the harmful environment persisted, forcing Ava to adopt early survival strategies such as suppressing her needs and striving to remain invisible to avoid triggering the volatile responses of her caregivers. Both her mother's harsh inconsistency and her father's neglectful warmth meant Ava grew up feeling frightened, anxious, unprotected, and alone.

#### 2.3.2 Key Fears

Ava's early traumatic experiences shaped her self‐perception and relational style, leading her to internalize a persistent sense of shame and unworthiness. Key fears included being invisible, inherently flawed, unlovable, and ultimately abandoned. Her internal narrative (“I'm a bad mother,” “I don't deserve good things,” “If I get better, I'll be alone”) was accompanied by heightened sensitivity to perceived rejection or criticism, particularly from her children. She lived in a near‐constant state of interpersonal vigilance, anticipating emotional danger and fearing becoming a burden.

#### 2.3.3 Protective Strategies

Ava appeared to live in a persistent state of anticipatory threat, characterized by hypervigilance to rejection, rapid escalation of self‐criticism, somatic tension, and difficulty tolerating emotional closeness. Ava also experienced intense fear of interpersonal threat, sadness related to attachment loss, and anxiety associated with hypervigilance towards possible judgment. Many of her coping strategies functioned to reduce this relational alarm and minimize rejection. Internally, she suppressed emotions, disconnected from painful memories, and identified with labels such as “damaged.” Historically, she relied on substances to numb shame, anxiety, loneliness, and intrusive memories, while seeking external validation to feel “seen.” Interpersonally, she engaged in people‐pleasing, reassurance‐seeking, and conflict avoidance. She often overcompensated by striving to appear competent, socially successful, with perfectly behaved children.

#### 2.3.4 Fragmented Trauma Memories

Ava's trauma memories of sexual abuse and emotional neglect, were conditioned to extreme states of threat and emotional isolation. These embodied memories remained fragmented, sensory‐laden, and fused with self‐blame. Without sufficient compassionate context at the time of trauma, she interpreted her freeze responses as personal failure. Following cessation of substance use, previously suppressed memories resurfaced with intensity, leaving her overwhelmed and urgently seeking reassurance. These flashbacks were not only re‐experiencing of traumatic incidents, but reactivation of shame‐based meanings.

#### 2.3.5 Unintended Consequences

Ava's survival strategies carried significant relational and psychological costs. Emotional suppression, self‐criticism, and substance use contributed to strained family relationships, periods of isolation, and fluctuating stability in parenting. Although these strategies initially functioned to reduce threat and manage overwhelming affect, they reinforced shame and disconnection over time. Her reliance on appeasement and reassurance‐seeking, learned in childhood as a means of staying safe, often led her to subjugate her own needs. This maintained relational insecurity and heightened vigilance to signs of rejection. These patterns were understandable adaptations to early attachment threat, yet they perpetuated her chronic relational alarm in adulthood.

#### 2.3.6 Ava's Self to Self‐Relating

Ava's internal critic was harsh, dominant, and punitive. She described it as “brutal but protective,” suggesting ambivalence about relinquishing it. The critic frequently labeled her as “too needy,” “not good enough,” “disgusting,” and “deserving of rejection.” Although experienced as hostile, its function appeared protective: it attempted to pre‐empt external criticism by attacking the self, first.

This internal hierarchy mirrored her early relational experiences, in which submitting to powerful others increased safety. The critic maintained emotional containment and a sense of control, but at the cost of reinforcing shame and hopelessness. This formulation directly guided the sequencing of intervention, with initial focus on reducing relational threat and building compassionate regulation capacities before engaging in trauma memory processing.

### 2.4 Course of Treatment

Following Ava's assessment her diagnosis of CPTSD was confirmed and her treatment goals were identified as: (1) to be able to live with past traumatic experiences and not have them influence everything in her life, (relationships, choices, and emotional responses); (2) to address Ava's relationships with her siblings and children and be able to respond to interpersonal interactions with compassion; (3) to make sense of her emotions and respond to how she is feeling with compassion. Ava also explained that she would like to improve her emotional literacy, improve her self‐worth and calm her mind; (4) to make sense of past traumatic experiences, understand her responses, and to get some form of closure; (5) to reduce her flashbacks and intrusive memories related to the abuse; and (6) to process grief related to extensive losses experienced throughout her life.

Based on her formulation, treatment prioritized addressing Ava's chronic threat activation and shame‐based self‐relating through a phased, group CFT approach to help her work towards her therapy goals. This consisted of a compassionate resilience phase, delivered as a closed 12 week group, with each weekly session lasting 2 h. The group commenced with six people; however, due to clinical considerations, two individuals transitioned to an individual phased treatment pathway after the group began. Following completion of the group, Ava began weekly individual trauma processing sessions, having 28 sessions with each lasting approximately 90 min. Lastly, Ava joined an 8‐session, pilot group which focused on enhancing life with compassion. Each session lasted 90 min.

#### 2.4.1 Phase 1: Compassionate Resilience Group (12 Group Sessions)

Ava was tentative about joining the group, feeling ashamed and worried that other members would judge her. In early sessions, she would sometimes go bright red, appear visibly distressed, and turn her camera off, particularly in response to the psychoeducation about attachment and parenting. The facilitator became concerned that her shame might prevent her from returning, as it felt overwhelming and at times unreachable by the group process. Ava later shared that she had nearly not attended on several occasions. When she disclosed this, the facilitator invited other group members to respond by sharing their own common experiences of shame and doubt. They emphasized how important she was to the group and how grateful they were that she had returned. Ava often reacted with further shame, saying, “you're just saying this to make me feel better, you don't really mean it.” The facilitator gently explored her fears: “What would happen if you allowed yourself to accept the comfort from the group, because they care about you? Let's see if we can stay with this for a moment and use our breath to steady us. What do you notice arising in your body as you rest into this?” When Ava allowed herself to pause, sadness would emerge, an emotion she found difficult to tolerate.

The facilitator introduced psychoeducation about loss and trauma, emphasizing the importance of honoring pain through grieving and allowing sadness and anger to surface as shame was met with compassion. The group explored how this emotional processing formed part of healing. The facilitator also highlighted Ava's courage in returning each week and her willingness to engage with her suffering. Ava stated she was determined to “do things differently this time.” There were moments in which Ava interpreted neutral group interactions as signs of disapproval. On one occasion, after another member spoke confidently about progress, Ava became withdrawn and later described feeling “behind” and “exposed.” This activated familiar rank‐based comparisons and fears of inferiority. The facilitators gently named this process and explored it within the group, helping Ava differentiate present‐moment experience from historical patterns of social threat. As the group progressed, de‐shaming psychoeducation and shared experiences increased Ava's compassionate awareness of her suffering. She began to recognize that her interpersonal style, shame, and guilt were understandable consequences of trauma rather than personal failings.

Alongside this relational work, Ava learned to settle her physiology through mindful attention, soothing rhythm breathing, and compassionate imagery. These practices aimed to stabilize physiological arousal and increase tolerance for distress before trauma processing. She was initially frightened of feelings such as sadness and anger, as they evoked loneliness and fear. She realized that she had rarely been comforted as a child and had learned to avoid emotional pain. Gradually, she developed greater tolerance for sadness and began grieving past parenting behaviors with compassionate understanding. Her narrative shifted from “I failed” to “I did the best I could.” The group helped her see that the traumatized version of herself had not intended to hurt her children. Her emerging compassionate self, wanted to engage with the suffering caused and make reparation. A turning point occurred when she apologized to her son for past behaviors and was met with forgiveness. This allowed her shame and self‐blame to soften into care and connection.

Ava's compassionate practice deepened through imagery exercises. She developed an internal compassionate scene (featuring Yoda, Bagpuss, and the Cheshire Cat) representing unconditional care and warmth. When she brought this scene to mind, she experienced embodied safeness and connectedness. She used this during a family outing when she felt a surge of perceived rejection from her children. Noticing the panic, she slowed her breathing, visualized her compassionate scene, and asked herself, “what would be a compassionate lens on this situation?” Shifting her physiological state enabled new insight and emotional regulation. Over time, Ava's language became more compassionate, and she became an emotionally generous group member, offering support to others using her own breakthroughs as learning examples. By the end of Phase 1, she described a noticeable internal shift: feeling more stable, less governed by shame, and increasingly able to hold her pain with warmth. This emerging compassionate resilience and strengthened self‐worth indicated readiness for *Phase 2* trauma memory work in individual therapy.

#### 2.4.2 Phase 2: Working with Trauma Memories Individual Sessions 1–28

Following the group phase, Ava began individual sessions with the aim to develop a compassionate, integrated narrative of her trauma history. Before this work began, the therapist spent a few sessions developing CFT formulation (see Section 2.3) to help Ava understand her difficulties. Drawing on de‐shaming psychoeducation from *Phase 1*, Ava began to make sense of her distress, not as a personal failing, but as an understandable outcome of how her mind had evolved, adapted to threat, trauma and social experiences. She began to appreciate the tragedy of her childhood and realized it was through no fault of her own, she was mistreated by others. The formulation helped her make sense of her relational world, providing insight into the unintended consequences of her best efforts to function in her social world.

Following this, Ava and her therapist mapped a trauma timeline, outlining the chronology of relational traumas, as well as positive experiences and areas of resilience (Lee and James 2012). Developing a trauma timeline supports integration of fragmented memories, helps distinguish past from present, and provides a coherent framework for compassionate meaning‐making. Ava was hesitant to see her story visually represented and feared the therapist would judge her, as she disclosed painful material. After sessions, she would “beat herself up” for “revealing too much,” returning to appeasing and self‐blaming patterns to preserve connection. The intensity of Ava's shame and self‐loathing evoked strong responses in the therapist. There were moments of internal pull to reassure or counter her self‐criticism quickly, particularly when Ava described herself as “disgusting” or “beyond help.” The therapist recognized that moving too rapidly to soothe risked reinforcing Ava's belief that her distress was intolerable or needed fixing. Slowing the pace, tolerating, *increasing window of tolerance* and maintaining a steady, compassionate presence became central relational interventions to maintain emotional regulation and reflective capacity. At times, the therapist also experienced doubt about whether the work was progressing, particularly when Ava withdrew or intensified self‐attack after sessions. These moments required reflective supervision and careful attention to the therapeutic alliance, reinforcing the importance of relational attunement, alongside technique (Matos and Dimaggio 2023).

The therapist focused on cultivating emotional safeness by modeling compassion and gently countering Ava's threat‐based expectations of the therapist's mind. Drawing on the formulation, they explored how self‐blame functioned as a safety strategy. When Ava stated, “I don't think I'm worthy,” the therapist invited her to slow down: “Let's just slow things down, use your breath, bring your compassion scene to mind and notice what happens in your body when you allow yourself to accept my compassion for you. What would happen if we discovered this was not your fault.” Tolerance for compassion was paced carefully, as relational alarm was easily activated. Alongside experiential practices, cognitive reappraisal of shame‐based meanings were supported through compassionate inquiry based on knowledge, understanding and wisdom.

As exploration deepened, Ava revisited key relationships, including her mother's aggression and inconsistency, and her father's volatility and absence. She began to appreciate how scared, lonely and unprotected she was as a child. She realized why she didn't/couldn't tell her parents about the abuse for fear that they wouldn't believe her or it would make things worse. She began to realize that this was not her fault. At this stage, they agreed to work directly with two distressing flashbacks, but revisiting memories of neglect and abuse quickly reactivated a powerful inner critic (“I'm disgusting,” “I must have wanted it”). The therapist initially used the group as a compassionate reference point (“What would the group say to you now?”), but the critic remained ruthless and relentless.

During one session, after recounting a painful memory, Ava became quiet and avoided eye contact. When asked what was happening, she said she felt the therapist was “probably thinking I'm weak.” The therapist moved to reassure her, but Ava withdrew further, stating reassurance felt “like you're just saying that because you have to.” This moment highlighted how rapidly relational alarm was activated. The therapist slowed the pace, acknowledged that expecting judgment made sense given Ava's history, and validated the difficulty of trusting benevolence. Naming the dynamic appeared to reduce tension, and Ava later reflected that having her fear understood felt different from being reassured. Yet the inner critic remained punitive and shaming, and they both agreed to pause trauma memory work and focus more directly on the inner critic and fears of compassion.

##### 2.4.2.1 Working with the Inner Critic

**Figure 4:**
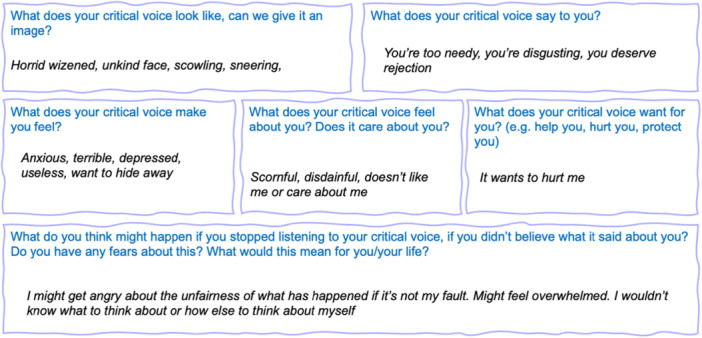
The forms and function of the self‐critic exercise for Ava.

Ava's fear of compassion was deeply rooted: “If I let myself feel worthy, I'll get hurt again…I deserve to be punished.” She also feared losing her critic, asking, “Who would I be without this?” The critic told her she was “too needy,” “not good enough,” and “deserving of rejection.” Although harsh, it functioned as a protective mechanism, attempting to maintain control and pre‐empt external attack. The therapist approached self‐criticism gradually, validating its protective intent while using the “forms and functions of self‐criticism” exercise (Figure 4) to externalize and examine it. By identifying its threat‐based origins in childhood, Ava began to understand the critic as a safety strategy rather than her identity. Over time, she differentiated from these internalized voices, stating, “This is how I was made to think about myself by other people, it's not who I am.”

Although Ava had previously used her “compassion scene” during Phase 1, her powerful critic seemed to sabotage access to it. It took time to revisit and develop whilst working alongside Ava's fears of compassion. This time, her “perfect nurturer” emerged as a pink fluffy bomber jacket hanging on a hook. She described being able to see it but not reach it: *I can see the jacket, but I can't get it off the hook*, seemingly symbolizing the distance between her and self‐compassion. The therapist supported gradual tolerance of this imagery, encouraging grounding and mindful attention to bodily sensations as she imagined reaching for the jacket. *Let's just stay with that feeling and notice your breath, use your grounding. Just allow yourself to sit with your need for the jacket and its comfort, what does this feel like? What would happen if you allowed yourself to reach it?* Over time, Ava discovered that using soothing rhythm breathing and compassionate intent, she was able to access the jacket: *When I pause and breathe, it becomes easier to reach it.* The image became a reliable regulator and compassionate anchor.

Ava was particularly troubled by feelings of shame and guilt over past alcohol misuse and the impact of this on children. Using breath, imagery and compassionate inquiry (*What would your children want you to know now?*), Ava began to tolerate their current love and trust. From a compassionate state of mind, she developed understanding toward her past self, allowing shame to shift toward reparative guilt. She chose to apologize to her children without self‐condemnation and in this instance, she was met with forgiveness, as they understood what a traumatic childhood she had endured.

##### 2.4.2.2 Using Chair Work to Access Compassion for Ava's Younger Self

Ava struggled to feel warmth toward her younger self. To address this, the therapist introduced chair work to facilitate direct compassionate engagement with the child who had experienced trauma and neglect. A chair was designated for her “child self,” and Ava was invited to sit opposite it and visualize her younger self there. Before speaking, she primed her compassionate motivation using grounding, breath, and the image of her pink bomber jacket. When asked what young Ava needed to hear, she initially froze, staring blankly at the empty chair. Shame immobilized her and with gentle prompting from the therapist, “What does young Ava need to hear? How can you help her feel safe?” she eventually said, “No child is nobody. No child doesn't matter. You matter too.” The therapist invited her to repeat these words slowly, using a warm and caring tone, allowing their impact to settle in her body. As she did so, she noticed resistance and the re‐emergence of the critic, telling her she did not deserve to speak to herself this way.

The therapist encouraged Ava to bring her attention back to her compassionate motivation, to hold the perfect nurturer in mind, use her breath to settle her threat response and notice sadness arising for her younger self. As she connected empathically with that pain she became overwhelmed by distress at not having been able to save the child. At this point she wanted to stop the exercise and said it wasn't “working.” The therapist also wondered whether the exercise was intolerable and if they should stop it. The therapist became aware of wanting to avoid upsetting Ava and recalled supervision about her transference experience with Ava. She asked herself, “What does Ava need to feel safe in this moment?.” Surmising that Ava needed to grieve for this experience, of not being protected, through no fault of her own, she gently encouraged Ava to bring her attention back to her compassionate motivation. Ava wanted to help her younger self, and to allow feelings of sadness and anger to emerge. She normalized these feelings as important and part of healing. Ava began to realize that she found it more helpful to think about her younger self as part of adult self and this led to her saying, *I can keep myself comfortable and safe now. I didn't have the capacity to look after her then, but I can nurture her now*. It marked a turning point in her ability to offer warmth inwardly, to sit with her vulnerability and take care of it.

##### 2.4.2.3 Returning to Work on the Flashbacks

By Session 24, foundational shifts were evident. Ava reported living in a “calmer mind,” starting to “like myself,” and being “not scared of tomorrow.” She no longer blamed herself for childhood harm or past substance use. Her capacity to grieve lost years, with compassion, to feel sadness and rightful rage, enabled her to tolerate and regulate affect better. Her self‐loathing lessened as did some of her interpersonal difficulties. She began to appreciate the tragedy of her past and asserted her entitlement to feelings without being ruled by guilt and shame. She was fundamentally reshaping her relationship to her past.

At this stage, they agreed to revisit two residual and persistent flashbacks from her childhood. Imagery rescripting (Arntz 2025) was used to update the emotional meaning of these memories from shame and self‐blame toward compassionate understanding. Ava described a memory of being abused by her stepfather, in a public place. She could hear people around her but felt trapped, unseen, frozen and powerless. For years, she believed it was her fault for not shouting for help. The therapist provided psychoeducation about automatic freeze responses under prolonged threat, helping de‐shame her immobility. She realized that, through no fault of her own, she had been frozen to the spot and speechless with fear. Collaboratively they explored how to rescript the memory in ways that made her feel empowered & protected. Ava spontaneously brought her perfect nurturer to mind and wondered what she might say to her, *You're not alone now. This wasn't your fault. You were just a child*. Ava also imagined herself as an adult, large and towering over the perpetrator, making him seem small and powerless. This worked better for her than imagining herself as a child being protected by her adult self. Once they worked out the re‐script, they primed Ava's compassionate state of mind, through soothing rhythm breathing and imagery. She was invited to bring the original memory to mind but this time re‐imagine herself as an adult, big and powerful, and able to protect herself from harm. She visualized herself looming over the perpetrator, and said, *He's the one quivering now, not me. He's the one that's done wrong, it's his shame*. Her therapist encouraged her to stay in the memory, with this image of herself, transforming her sense of vulnerability to that of embodied empowerment. She then asked Ava to bring her perfect nurturer to the scene and listen to the words of comfort offered. *You're not alone now. This wasn't your fault. You were just a child—you didn't know, you hadn't been taught how adults should treat children. You hadn't experienced good care from adults.* The therapist encouraged Ava to use a low, soft, caring, voice tone as she said those words and, to stay with the emergent feelings of sadness and anger. Ava was beginning to honor the pain and loss associated with these experiences, processes previously blocked by states of shame and self‐blame.

Re‐scripting Ava's trauma memories helped her begin to meet her unmet childhood need for protection and care. She felt a sense of connectedness in her mind as she imaged comfort from her perfect nurturer. She described a post‐session shift, *It's like I've been falling my whole life and now I've landed on something solid, now I can rely on myself, I don't need to carry the burden now*.

##### 2.4.2.4 Multiple Selves Exercise: Unpacking “The Sting” of Not Being Protected

Although flashbacks were no longer intrusive following rescripting, Ava described a residual emotional “sting.” The therapist suggested further chair work to explore unresolved feelings, particularly toward her mother for failing to protect her. She placed four chairs in a circle representing sad‐self, anxious self, angry‐self, and compassionate‐self. She invited Ava to sit in each chair, finding her voice and feelings towards her mother from that perspective (e.g., “what does sad‐self want to say to your mother?”). The therapist paid particular attention to the parts of self Ava found hard to access and express, such as feelings of sadness and anger. With encouragement and permission, she was able to say, *She didn't protect me. She let me down*. Through guided reflection in the compassionate chair, at the end of the exercise, Ava developed a compassionate perspective that acknowledged both her mother's limitations and her responsibility: *She didn't mean to harm me. But she should have protected me. She didn't know how, she didn't have the tools either. She didn't have the knowledge; she didn't have the language. She didn't have anyone to back her either. She was powerless too and this wasn't her fault. She did the best with what she had.*

The compassionate perspective allowed Ava to acknowledge both the harm and the complexity of her attachment history, an essential act of integration. The chair work allowed for emotional differentiation, development of compassionate understanding without minimizing the harm caused and adaptation to relational trauma by holding the complexity and ambivalence of her feelings towards her mother. At a later stage Ava reflected on feeling sad for her mother now. She no longer felt the sting, but a sense of sadness for how her mother was treated as a child, and as well as for herself. This helped Ava bring up and remember some good memories from her childhood and she felt those were becoming “bolder” in her mind. She commented that she felt she gained more connection with her mother, when she was freed from blaming her.

#### 2.4.3 Phase 3 Enhancing Life with Compassion

Following individual therapy, Ava attended 7 of 8 sessions of the online Enhancing Life Group pilot to consolidate CFT skills. The program focused on identifying personal values, taking compassionate action, and relapse prevention. Ava engaged fully, sharing reflections, and applying concepts in her daily life, such as using a “compassionate blanket” for distress and making healthier food choices as self‐care. She also successfully joined a peer support group and an art group, boosting her confidence and self‐esteem. By the end, she demonstrated a commitment to sustaining her growth and compassionate relationships by agreeing to stay connected with group members.

### 2.5 Outcome and Prognosis

Ava completed self‐report outcome measures at five timepoints. By the end of therapy, she showed clinically significant reductions in PTSD and CPTSD symptoms (PCL‐5: 63 → 11; ITQ: 39 → 7) and trauma‐related shame (TRSI total: 56 → 1). She also reported marked reductions in self‐criticism, particularly “hated self” scores, and increased self‐reassurance and wellbeing. A summary of these scores can be found below in Table 1. Functionally, Ava described feeling *more me*, less dominated by shame, more emotionally regulated, and better able to connect with her children. She described no longer identifying primarily as *traumatized* and no longer relied on reassurance‐seeking, self‐harm, or substances to manage distress. Instead, she drew on compassionate imagery, such as her *pink bomber jacket*, and her compassionate motivation to navigate challenges.

**Table 1:** Ava's outcome measures at five timepoints.

Scales						RCI	
	T1	T2	T3	T4	T5	T2 to T3	T2 to T4
PCL‐5	63	66	61	11	5	−0.57	−6.23*
ITQ	39	44	36	7	3	−0.92	−4.24*
PHQ‐9	15	23	16	11	14	−2.03*	−3.48*
FSCRS‐ Inadequate self	31	35	25	6	11	−1.58	−4.60*
FSCRS‐ Reassured self	7	10	19	17	17	1.82	1.41
FSCRS‐ Hated self	14	15	10	0	4	−1.64	−4.93*
WSAS	31	29	27	12	16	−0.34	−2.89*
WEMWBS	35	30	43	49	44	2.56*	3.74*
DEMO	75	101	111	47	65	1.17	−6.34*
TRSI‐ Total	56	65	32	1	8	−3.40*	−6.60*
TRSI‐ Internal	32	33	20	0	6	−3.61*	−9.17*
TRSI‐ External	24	32	12	1	2	−6.60*	−10.23*
CEAS‐ SC Engagement	—	27	25	42	50	– 0.67	4.99*
CEAS‐ SC Action	—	10	18	29	33	1.50	3.56*
CEAS‐ CO Engagement	—	16	35	38	31	4.19*	4.86*
CEAS‐ CO Action	—	8	22	30	32	4.72*	7.43*
CRS	—	39	58	84	90		

*Note:* T1, Assessment; T2, Start of CRG; T3, End of CRG and start of individual therapy; T4, End of therapy, T5, Follow up.

Scales are the following: PTSD Checklist for DSM‐5 (PCL‐5); International Trauma Questionnaire (ITQ); Patient Health Questionnaire‐9 (PHQ‐9); Forms of Self‐Criticism and Self‐Reassuring Scale (FSCRS); Work and Social Adjustment Scale (WSAS); Warwick Edinburgh Mental Wellbeing Scale (WEMWBS); Dissociative Experiences Scale, Oxford (DEMO); Trauma Related Shame Inventory (TRSI); Compassionate Engagement and Action Scales (CEAS)—Self Compassion (SC) and Compassion from Others (CO); Compassionate Resilience Scale (CRS).

– indicates that a questionnaire was not completed at this timepoint.

A statistically reliable change is indicated with *. As the CRS is not yet validated, reliable change was not calculated for this measure. Reliable change was calculated comparing scores between start of therapy and end of the CRG and start and end of therapy.

She described handling family criticism with greater clarity and less emotional fusion, reframing past events through a compassionate lens, and differentiating feelings from identity (e.g., “I feel disgusted” rather than “I am disgusting”). She reported confidence in facing future life changes and a willingness to live by her values. She felt more able to trust herself: *My past is there but it doesn't control me anymore. I've learnt how to be kind to myself and to others around me. I've learnt to take things in my stride, I don't stress or overly worry about the trauma, I just feel comfortable, I feel comfortable in myself, I have a sense of liking myself and don't need to remind myself of this all the time, I feel OK now, I'm happy with this. I'm content*. At follow‐up, Ava maintained these gains, remained abstinent from substances and self‐harm, and continued to engage in valued community activities.

## 3 Clinical Practices and Summary

This case highlights the potential utility of phased trauma‐focused CFT for CPTSD, particularly when pervasive shame, self‐criticism, and fears of compassion are central maintaining processes. Ava's presentation was chronic and severe, and she had many years of contact with mental health services prior to being referred to a specialist service. Ava's improvements suggest that cultivating compassionate motivation prior to intensive trauma processing may enhance emotional tolerance and, support shifts in trauma‐related meaning. However, this is a single case conducted within a highly specialized and well‐resourced service. Ava required a high level of input across group and individual phases, which allowed repeated opportunities to address fears of compassion and relational threat as they emerged. Deeply entrenched self‐loathing and beliefs of not deserving compassion present considerable challenges in trauma focused therapy as the role of self ‐blame is a well‐documented maintenance process in CPTSD (Harman and Lee 2010). Also, there are challenges within interpersonal processes and relational considerations that need understanding, formulating and addressing. Having the time and space to appropriately address these challenges as they occur is crucial in CPTSD, as relationship distrust and discord are central features of the disorder (Popolo et al. 2024). That said, there is an increasing pressure on clinicians to deliver therapies across shorter durations and session numbers. The intensity and duration of intervention may not be feasible in all settings, and therapist expertise in both trauma work and CFT likely contributed to outcome. Therapists in private practice might have pragmatic difficulties coordinating multiple phases, including groups. In settings where group delivery is not feasible, key elements of this phased model may be adapted for individual therapy.

## Ethics Statement

The case‐study was approved by the ethical review board of University of Queensland, and the patient gave written informed consent.

## Conflicts of Interest

James Kirby is the co‐author of “Essential of Compassion Focused Therapy: A Practice Manual for Clinicians” which he obtains royalties. The other authors declare no conflicts of interest.

## Data Availability

Data can be obtained through contacting the authors.
